# Mapping potential effects of proposed roads on migratory connectivity for a highly mobile herbivore using circuit theory

**DOI:** 10.1002/eap.2207

**Published:** 2020-08-18

**Authors:** Timothy J. Fullman, Ryan R. Wilson, Kyle Joly, David D. Gustine, Paul Leonard, Wendy M. Loya

**Affiliations:** ^1^ The Wilderness Society Anchorage Alaska 99501 USA; ^2^ Gates of the Arctic National Park and Preserve Arctic Inventory and Monitoring Network National Park Service Fairbanks Alaska 99709 USA; ^3^ Grand Teton National Park National Park Service Moose Wyoming 83012 USA; ^4^ Science Applications U.S. Fish and Wildlife Service Fairbanks Alaska 99701 USA; ^5^ Science Applications U.S. Fish and Wildlife Service Anchorage Alaska 99503 USA; ^6^ Present address: Marine Mammals Management U.S. Fish and Wildlife Service Anchorage Alaska 99503 USA

**Keywords:** Arctic, caribou, Circuitscape, development, disturbance, landscape resistance, management, migration, *Rangifer tarandus*

## Abstract

Migration is common worldwide as species access spatiotemporally varying resources and avoid predators and parasites. However, long‐distance migrations are increasingly imperiled due to development and habitat fragmentation. Improved understanding of migratory behavior has implications for conservation and management of migratory species, allowing identification and protection of seasonal ranges and migration corridors. We present a technique that applies circuit theory to predict future effects of development by analyzing season‐specific resistance to movement from anthropogenic and natural environmental features across an entire migratory path. We demonstrate the utility of our approach by examining potential effects of a proposed road system on barren ground caribou (*Rangifer tarandus granti*) and subsistence hunters in northern Alaska. Resource selection functions revealed migratory selection by caribou. We tested five scenarios relating habitat selection to landscape resistance using Circuitscape and GPS telemetry data. To examine the effect of potential roads on connectivity of migrating animals and human hunters, we compared current flow values near communities in the presence of proposed roads. Caribou avoided dense vegetation, rugged terrain, major rivers, and existing roads in both spring and fall. A negative linear relationship between resource selection and landscape resistance was strongly supported for fall migration while spring migration featured a negative logarithmic relationship. Overall patterns of caribou connectivity remained similar in the presence of proposed roads, though reduced current flow was predicted for communities near the center of current migration areas. Such data can inform decisions to allow or disallow projects or to select among alternative development proposals and mitigation measures, though consideration of cumulative effects of development is needed. Our approach is flexible and can easily be adapted to other species, locations and development scenarios to expand understanding of movement behavior and to evaluate proposed developments. Such information is vital to inform policy decisions that balance new development, resource user needs, and preservation of ecosystem function.

## Introduction

Migration is an important process for many species, with benefits including access to spatiotemporally varying resources and reduction of predators and parasites (Dingle and Drake 2007, Southwood and Avens 2010, Avgar et al. 2014, Brönmark et al. 2014). Migratory species contribute to ecological processes such as nutrient transport between diverse systems and temporary alteration of local trophic interactions (Brodersen et al. 2011, Bauer and Hoye 2014, Brönmark et al. 2014). These environmental effects also influence human food security, disease dynamics, and human–wildlife conflict and coexistence (Graham et al. 2009, Ziv et al. 2012, Liu et al. 2013, Hassell et al. 2017). Yet, migratory movements may be altered due to human development (Wilson et al. 2016) and long‐distance migrations are becoming increasingly imperiled across the globe due to development and habitat fragmentation (Berger 2004, Bolger et al. 2008, Wilcove and Wikelski 2008). Impediments to migration have often led to significant declines in populations (Bolger et al. 2008). Thus, improved understanding of migratory behavior not only can increase theoretical understanding of migration ecology, but also has implications for conservation and management of migratory species, allowing identification and possible protection of key seasonal ranges and migration corridors (Harris et al. 2009).

Land‐use change and landscape fragmentation are increasing globally (Ibisch et al. 2016, Torres et al. 2016), and there is a need for adequate planning to minimize negative environmental effects of development activities. This is all the more important because adverse effects of development projects may persist for years, despite intensive mitigation efforts (Sawyer et al. 2017). Environmental assessments, such as those conducted under the National Environmental Policy Act (NEPA) in the United States, often rely on analyses of expected outcomes of proposed development activities on species, habitats, and people. In many cases, however, it can be challenging to predict the ecological effects of proposed development, highlighting the need for improved analyses to aid in environmental assessments. It is also increasingly recognized that natural and human systems cannot be managed separately, but rather are coupled together as wildlife and other natural systems increasingly interact with, affect, and are influenced by people (Liu et al. 2007, Carter et al. 2014).

We predict future effects of development by analyzing resource selection and resistance to movement across an entire migratory path from both anthropogenic and natural environmental features. We then use the resulting estimates of migratory resistance to project the expected impacts of potential development on migratory species and human resource users. While many connectivity studies for animal migration define resistance as the inverse of habitat suitability (Bond et al. 2017), this assumption has been questioned (Keeley et al. 2016, 2017, Ziółkowska et al. 2016). We directly test the assumption of inverse habitat suitability by exploring the relationship between habitat suitability and landscape resistance in a scenario testing framework. Scenario testing has long been a useful tool in conservation planning (Peterson et al. 2003), and we use it here by representing contrasting alternatives for responses of animals to their environment (sensu Cushman et al. 2006). Our scenarios evaluate alternative resistance landscapes using GPS‐derived locations to select between alternatives.

We demonstrate the utility of our approach by applying it to one of the world’s longest terrestrial migrants: barren ground caribou (*Rangifer tarandus granti*) of the Western Arctic Herd (WAH) in northwestern Alaska (Joly et al. 2019). Caribou, along with related reindeer (*R. t. tarandus*), are the most abundant large terrestrial herbivore in the circumpolar arctic (Bråthen et al. 2007), spanning North America, Europe, and Asia (Festa‐Bianchet et al. 2011, Mallory and Boyce 2018). Caribou are renowned for their long‐distance migrations, covering hundreds to thousands of kilometers each year in some of the longest overland movements in the world (Fancy et al. 1989, Joly et al. 2019). During these movements, caribou influence vegetation and nutrient patterns through grazing and trampling (Stark et al. 2015, Heggenes et al. 2018) and serve as a prey species for brown bears (*Ursus arctos*), wolves (*Canis lupus*), and other predators (Reynolds and Garner 1987, Dale et al. 1994, Ballard et al. 1997, Mowat and Heard 2006, Magoun et al. 2018). Although widely distributed, many caribou and wild reindeer populations have faced large declines, likely due to global changes in climate and anthropogenic landscape change (Mallory and Boyce 2018).

Caribou play a central role in both food security and cultural well‐being of many indigenous groups across the circumpolar arctic (Bjørklund 1990, Berkes et al. 1994, Braem 2012). Subsistence hunting, fishing, and gathering are important as sources of nutrition and to support culture, identity, and customary and traditional ways of life (Lambden et al. 2007, Smith et al. 2009, Fall 2018). In northwestern Alaska, subsistence harvest contributes substantially to food security, providing between 180–450 kg of wild foods per person per year (Magdanz et al. 2011). Caribou comprise a large part of this harvest. One survey of six villages found that between 37–118 edible kilograms of caribou were harvested per person per year (Braem 2012), with an individual caribou yielding about 53 kg of edible meat (Braem et al. 2011).

To inform management of caribou and their harvest, we apply circuit theory combined with empirical location data and landscape alternatives, using the results to represent migratory movement and habitat selection of caribou across broad spatial scales relevant to managing human impacts. Circuit theory can model movement of organisms across landscapes under an analogy of electrical current flowing across a resistance surface (McRae et al. 2008). Under circuit theory, cells in a landscape are treated as nodes in an electrical circuit that are connected to their neighboring cells with resistors that have strengths based on the landscape’s resistance to animal movement. Current is passed through the circuit from a source location, across the resistance landscape, and to a grounded destination. The resulting current value in each cell is proportional to the number of times a random walker travelling from the source to destination would be expected to pass through that cell (McRae et al. 2008). The approach has been applied to connectivity and gene flow for a wide range of taxa and environments (McRae and Beier 2007, Lawler et al. 2013, Braaker et al. 2014, Koen et al. 2014). While some have suggested that cost‐distance approaches, such as least cost paths, may be more appropriate for modeling migratory connectivity (McClure et al. 2016), previous analysis of fall migratory movement for the WAH found that movement patterns better align with a random walk movement model, such as that underlying circuit theory (Fullman et al. 2017).

Using our combination of circuit theory, GPS telemetry, and landscape resistance scenarios, we analyze (1) which landscape features are selected or avoided at the scale of the entire migratory path of a caribou population; (2) the season‐specific relationship between relative habitat suitability and environmental resistance during caribou migration; (3) how migratory pathway suitability is predicted to be affected by a proposed network of roads; and (4) how road effects are expected to influence subsistence hunters that rely on caribou.

## Methods

### Study area

**Fig. 1:**
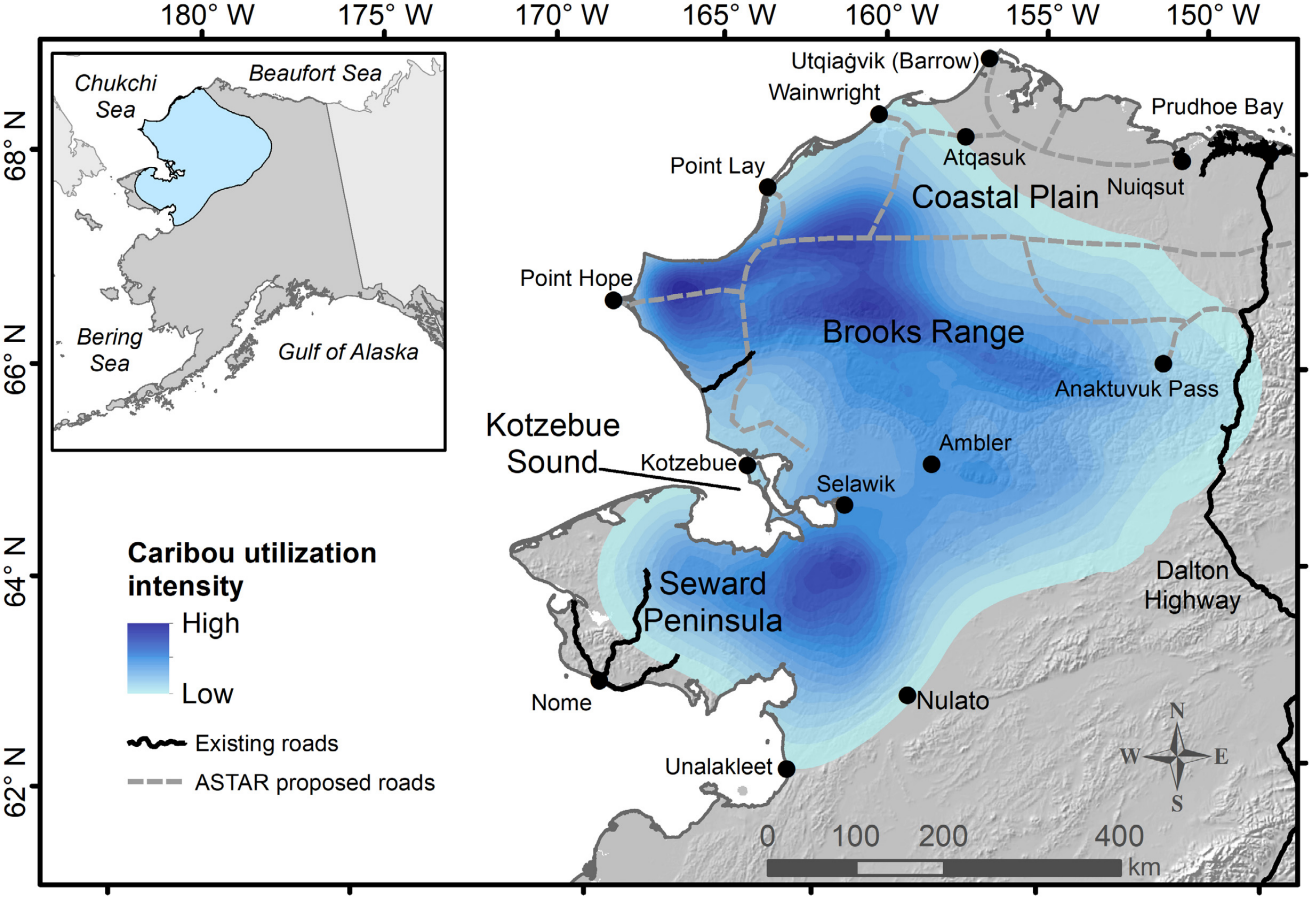
Western Arctic Herd range. Caribou migrate each fall to their winter range, concentrating in the dark blue region in the south. In the spring, they return to their calving range, indicated by the central dark blue region in the north. Existing gravel roads are sparse within the region but the Arctic Strategic Transportation and Resources (ASTAR) project proposes a potential system of roads across much of the herd’s northern range. Utilization intensity data provided by the Alaska Department of Fish and Game. ASTAR roads digitized from an Alaska Department of Natural Resources map.

The WAH is the largest caribou herd in Alaska, with ~244,000 individuals as of July 2019 (A. Hansen, *personal communication*). The herd occupies the northwestern portion of Alaska, covering a range of ~363,000 km^2^ (Fig. 1). Most caribou migrate north in the spring to calve and spend the summer on the northern coastal plain of Alaska and in the foothills and mountainous regions of the Brooks Range (Dau 2011). In the fall, most caribou migrate south to winter in the eastern Seward Peninsula, Nulato Hills, and upper Kobuk River (Joly et al. 2007, Dau 2013). The WAH is harvested for subsistence by Alaska Native and other hunters from over 40 communities in northwestern Alaska (Braem 2012), as well as by hunters from other parts of Alaska and outside Alaska, who jointly comprise about 5% of the annual harvest (Dau 2015).

### Caribou migration data

We recorded caribou migration paths using GPS telemetry data collected from 2009 to 2013. Adult female caribou were fitted with GPS telemetry collars (Telonics TGW‐4680; Mesa, Arizona, USA) as they swam across Onion Portage on the Kobuk River (Dau 1997, Joly 2011, 2011). Collars recorded location data every 8 h. The Animal Care and Use Committee of the Alaska Department of Fish and Game approved all animal handling under protocol #2012‐031R.

We determined start and end locations of migration for each caribou using net‐squared displacement (NSD; Bunnefeld et al. 2011), with fall and spring migration analyzed separately. In short, NSD describes the distance between a starting location and subsequent locations. For migratory species that feature clustered seasonal range use of repeated areas separated by persistent directed movement, NSD curves take on a characteristic shape in which values are initially low, increase rapidly during migration, level out, and then rapidly diminish. The start and end of the sharp increase and decrease in NSD values can be used to infer departure and arrival during migration (Bunnefeld et al. 2011). We used visual assessment of migration start and end from NSD plots (Mysterud et al. 2011) to avoid spurious results, as reported in previous studies (Mysterud et al. 2011, Bischof et al. 2012, Panzacchi et al. 2013).

### Identifying migratory habitat selection

Resource selection occurs at a hierarchy of scales (Johnson 1980) and habitat selection patterns may differ across scales (Boyce et al. 2003). Because our interest was in selection at the scale of the entire migratory path, to understand areas used for migration out of the total annual range, we conducted a population‐level resource selection function (RSF) analysis. We extracted fall and spring migration locations from the annual dataset using the migration start and end dates identified as described (see *Caribou migration data*). Generalized linear mixed effects (GLMM) models with random intercepts for year nested within each animal, run using the lme4 package (Bates et al. 2015) in R (version 3.6.1; R Core Team 2019), represented migration path selection by caribou separately for each season. We included 20 available points for each observed caribou location, based on a sample size sensitivity analysis (Northrup et al. 2013; Appendix S1). Available points were randomly generated across the study area, which we identified by a square bounding box slightly larger than the annual range of the herd (see Fig. 1 for annual herd range and Appendix S2: Fig. S1 for study area extent).

We investigated four environmental variables thought to influence caribou movement at broad scales: terrain ruggedness, major rivers, land cover, and existing roads (Appendix S2: Fig. S1). We calculated terrain ruggedness following Sappington et al. (2007), using three‐dimensional vector dispersion to account for heterogeneity in both slope and aspect as a representation of topographic variability. The analysis used an eight‐pixel moving window on a 60‐m digital elevation model (Gesch 2007). We centered terrain ruggedness values by subtracting the mean and dividing by the standard deviation to aid model convergence. We obtained locations of first‐order rivers, referred to here as “major rivers,” from the National Hydrography Dataset (NHD, *available online*)^8^ and converted their shapefiles to a raster using ArcGIS (ESRI, Redlands, California, USA). To represent broad land cover classes that might influence caribou migratory movements, we reclassified a 30‐m composite map of Alaska land cover (Boggs et al. 2014) into three classes: open areas, dense vegetation, and burned areas (see Appendix S2: Table S1 for crosswalk). While large spans of open ocean are substantial barriers, caribou will cross sea ice (Miller et al. 2005, Joly 2012), thus near‐shore coastal waters (within 3 km of the coast, plus all of Kotzebue Sound) that may at times be covered in sea ice were included as a fourth class: coastal waters (Appendix S2: Fig. S1). Open areas were used as the reference class in all RSF models.

Existing roads consisted of gravel and dirt roads including the Dalton Highway, roads associated with oil, gas and mining development, and roads extending from the town of Nome, Alaska (Fig. 1). We did not include small roads occurring within communities. To account for nonlinear responses of distance to road, we employed the approach of Carpenter et al. (2010, adapted from Nielsen et al. 2009). This featured an exponential distance decay function of the form e^‐α/^ *^d^*, where *d* was the distance from each used or available location to the nearest road in kilometers and α was allowed to vary between seasons. This resulted in values ranging between 0 and 1 (Appendix S2: Fig. S1). With little clear theoretical basis on which to select the α value, we tested a number of different α values as well as a linear relationship with distance to roads using univariate GLMM models, selecting among them using Akaike’s Information Criterion corrected for small sample size (AIC_c_; Burnham and Anderson 2002, Carpenter et al. 2010). We retained the best‐performing metric to represent the influence of roads on caribou migration (Carpenter et al. 2010) and included this metric in our model selection described in the following paragraph. Candidate models included α values every kilometer from 1–20, every 5 km from 25–50, every 10 km from 60–100, and every 50 km from 150–350 (Appendix S2: Tables S2, S3).

In an effort to align the functional grain (scale at which caribou may respond to landscape features or covariates) with the analysis grain (resistance surfaces) we chose to rescale environmental covariates to 1 km. This corresponds to the resolution at which road impacts on Alaskan caribou have previously been examined (Cameron et al. 1992, Joly et al. 2006, Johnson et al. 2020). This scale was also a compromise to achieve computational efficiency. Although we hypothesize that the functional grain may be larger than 1 km for some covariates, several studies have shown that when the analysis grain is finer than the functional grain, there is a small effect on the accuracy of resistance distance estimates (Cushman and Landguth 2010, Galpern and Manseau 2013). Investigation of variance inflation factors (VIFs) indicated collinearity was not an issue (all VIF values <1.1; Zuur et al. 2010, Dormann et al. 2013), so we created a set of candidate RSF models using a factorial combination of environmental variables, plus an intercept‐only model, resulting in 16 total models. We selected the top model for each season using AIC_c_ with the AICcmodavg R package (Mazerolle 2019). We evaluated predictive performance of the top models using *k*‐fold cross‐validation (Boyce et al. 2002, Johnson et al. 2006) by splitting the caribou location data for each seasonal migration into 10 parts, with 90% of the data used to train the model and 10% of the data withheld to test the model in each fold. We visualized the relative probability of selection by caribou with respect to distance from roads using model‐based parametric bootstrapping with the bootMer function in lme4 (Bates et al. 2015). For each season, 500 bootstrap simulations provided estimates of the mean response and 95% confidence interval of caribou relative selection with respect to roads. We held all other variables at their mean (terrain ruggedness) or baseline (river = 0, land cover = open areas) levels when creating these predictions.

### Resistance scenario testing

While resistance to animal movement is often assumed to be the inverse of habitat suitability, animals may display more complicated resistance relationships with their environment (Braaker et al. 2014, Keeley et al. 2017). We examined the relationship between relative habitat suitability and landscape resistance directly by analyzing five resistance scenarios (Table 1, Appendix S2: Figs. S2 and S3; Braaker et al. 2014). These ranged from having resistance be related only to distance (r0), to a positive relationship with habitat suitability (r1), to three variants of negative relationships between resistance and suitability, varying in the degree to which high resistance or low resistance predominates across the landscape (r2–r4; Table 1). Resistance values under each model, except the neutral (r0), were stretched to range between 1–1,000. It is the relative magnitude of resistance variables, rather than their specific values, that is informative when evaluating between alternatives (Cushman et al. 2006).

**Table 1:** Resistance scenarios used to test the relationship between relative habitat suitability and landscape resistance for migratory caribou in northwestern Alaska, USA.

Resistance scenario	Relationship	Formula
r0: Neutral	all equal	*r* = 1
r1: Positive linear	positive	*r* = hs
r2: Negative exponential	mostly low resistance	*r* = (1/hs)^2^
r3: Negative linear	negative	*r* = (1/hs)
r4: Negative logarithmic	mostly high resistance	*r* = ln(1/hs)

Notes

*r*, landscape resistance to movement; hs, relative habitat suitability. See Appendix S2: Figs. S2 and S3 for maps of resistance landscapes under each scenario for fall and spring migration, respectively.

We used circuit theory to investigate which resistance scenario best fit the data for WAH fall and spring migration routes. We modeled expected flow of caribou under the various resistance landscapes using Circuitscape (version 4.0.5; Shah and McRae 2008) in raster mode with pairwise calculation and connectivity calculated using a cell’s eight nearest neighbors. The start and end points in each caribou’s migration path (see *Caribou migration data*) defined source and ground locations, respectively, for electrical current density (i.e., “flow”) in the Circuitscape model. We generated cumulative maps of current flow for each caribou–season–year–scenario combination. See Data S1 for an example initiation file for Circuitscape.

Caribou GPS data allowed us to determine support among alternative resistance scenarios. We extracted current flow values produced by Circuitscape for each resistance scenario at all recorded locations along the migration path for an individual using the raster package (Hijmans 2019) in R. Summing these values yielded the total current flow for a given resistance scenario. We ranked the underlying resistance scenarios for each individual, with the highest summed current flow at observed caribou locations yielding the best ranking. We tabulated the number of times each resistance scenario appeared as a top‐ranked model to demonstrate the consistency of support for each resistance relationship across individuals, inferring greater confidence in resistance scenarios that consistently appeared in the top‐ranked models compared to a scenario more evenly spread among rankings. We mapped population‐level connectivity for fall and spring migration across the WAH range by summing the top‐ranked maps for each individual after standardizing each raster to sum to one. *K*‐fold cross‐validation with 10 folds indicated model performance for the population‐level connectivity layers. Example code for all analyses can be found in Data S1.

### Evaluating effects of a proposed road system

We used the observed responses of caribou to existing roads (see *Results*) to predict the potential effects of a proposed system of roads on the flow of caribou during migration. In 2017, the Alaska Department of Natural Resources initiated the Arctic Strategic Transportation and Resources (ASTAR) project. The project encompassed a three‐year effort to develop a strategic plan to re‐write existing management policy and support creation of roads and other infrastructure between communities and resource development areas (Mack 2017). Publicly posted maps depicted potential routes for ASTAR roads that cross the WAH range (Fig. 1). We downloaded and digitized the ASTAR proposal map (DNR 2017). This map was not intended to represent final locations of ASTAR roads, but rather represents one realistic depiction of the location of future roads in an area that currently is predominantly roadless. As such, it presented an opportunity to demonstrate the ability of our approach to examine effects of potential roads on caribou migration.

We generated a resistance landscape for each caribou using the top‐ranked scenario identified for that individual. In this resistance landscape, the season‐specific road selection distance decay functions and coefficients identified in the above analysis were applied to both existing roads and possible future roads (Appendix S2: Figs. S4 and S5). Similar to the creation of the population‐level connectivity maps described above, we ran Circuitscape on each of these resistance landscapes and summed the standardized results to represent population‐level connectivity in the presence of the proposed road system.

To examine the effects of potential roads on connectivity of migrating caribou and subsistence hunters, we compared current flow values near communities in the presence and absence of proposed roads. Point locations of communities within the range of the herd (Appendix S2: Fig. S6), buffered by 20 km, represented easily accessible caribou hunting areas. While subsistence hunting of caribou occurs across wide areas (Brinkman et al. 2014), analysis from western Canada reported preferences for and highest use of hunting near communities in areas accessible during day trips (Berman and Kofinas 2004). Variability exists, however, in area covered for subsistence harvest among different communities in northwestern Alaska and within communities, such as based on hunter age and gender (Satterthwaite‐Phillips et al. 2016). We extracted current flow values within the 20 km buffers from the population‐level current flow maps with and without possible roads included and calculated effect sizes for each community using raw score standardization, since our interest was in differences with and without possible roads (Morris and DeShon 2002). We used the thresholds suggested by Cohen (1992) to represent the approximate levels of effect.

## Results

Location data represented 57 caribou for fall migration and 70 caribou for spring migration. Collars provided one to four years of location information per caribou, resulting in 131 fall migrations and 153 spring migrations (referred to here as “individuals”). We did not conduct a study of collar location accuracy as part of this project, however, previous work with similar collars reported an average location error of 33 m (Joly 2005), well within the resolution of our covariate data.

Model selection analysis of the effect of roads on caribou locations indicated strong support for exponential decay over a linear relationship with distance (Appendix S2: Tables S2 and S3). For fall migration, a decay function of 60 km was most strongly supported, with ΔAIC_c_ > 2 compared to the next closest model and 87% of the Akaike weight (Appendix S2: Table S2). Results in spring were more evenly mixed, with alpha values of 12, 13, 14, and 15 km all within 2 AIC_c_ of the best‐supported model (Appendix S2: Table S3). We followed Carpenter et al. (2010) and retained only the best‐performing metric (α = 14) as a representation of the effect of roads on caribou migration in subsequent models for spring migration.

**Fig. 2:**
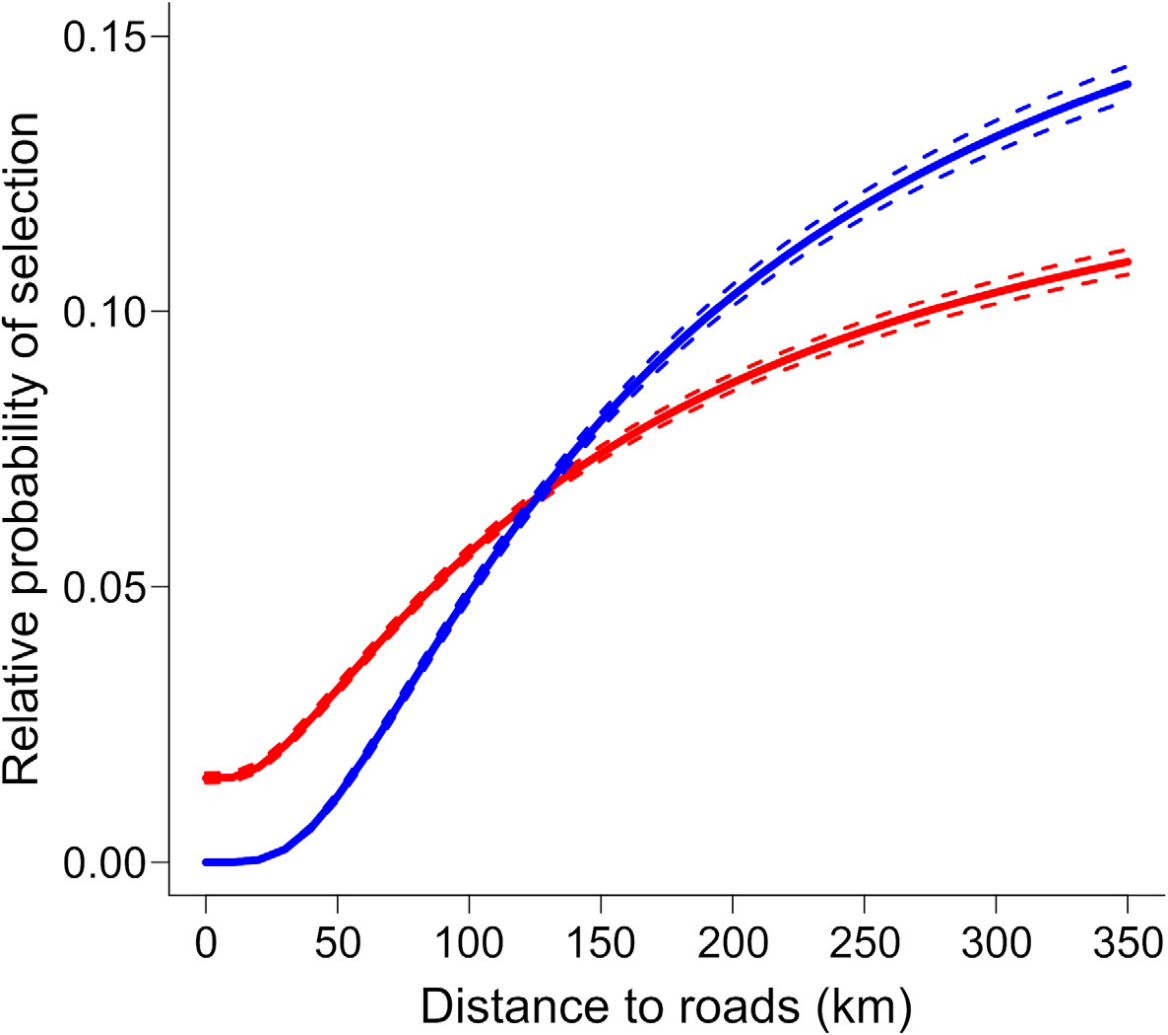
Relative probability of selection by caribou with respect to distance from roads for fall (red) and spring (blue) migration. Solid lines represent means and dashed lines 95% confidence intervals from 500 bootstrap simulations. Relative probability of selection was calculated with all variables other than distance to roads held at their mean (terrain ruggedness) or baseline (river = 0, land cover = open areas) levels.

Comparison of caribou resource selection models using AIC_c_ indicated strong support for the full model, containing all four environmental variables, in both fall and spring (Appendix S2: Tables S4 and S5). Resource selection patterns were similar in both seasons (Table 2). Caribou showed negative responses to increasing terrain ruggedness and the presence of major rivers, and showed greater avoidance of dense vegetation, burned areas, and coastal areas relative to open land cover types. Prediction of relative probability of selection at increasing distances from roads, with all other coefficients held at their mean or baseline values, indicated a low probability of selection near roads (Fig. 2). Road avoidance was stronger in spring compared to fall (Table 2, Fig. 2). All coefficients had 95% confidence intervals that did not overlap zero. *K*‐fold cross‐validation indicated high predictive performance of resource selection models in both seasons, as denoted by Spearman‐rank correlation ( ${\bar{\rho }}_{\text{fall}}=0.96$, ${\bar{\rho }}_{\text{spring}}=0.95$).

**Table 2:** Top model results for caribou resource selection during fall and spring migration.

Environmental variable	Fall		Spring	
	Estimate	SE	Estimate	SE
Intercept	−4.17	0.03	−14.04	0.21
Ruggedness	−0.22	0.01	−0.20	0.01
Major rivers	−0.56	0.04	−0.28	0.04
Distance decay from roads†	2.45	0.04	12.74	0.23
Dense vegetation‡	−1.17	0.02	−1.56	0.03
Burned areas‡	−0.26	0.07	−1.33	0.14
Coastal areas‡	−2.82	0.13	−2.69	0.14

† Distance decay followed e^‐α/^ *^d^*where *d* is distance in km and α = 60 in fall, 14 in spring.

‡ Selection reported relative to the open areas reference class.

**Fig. 3:**
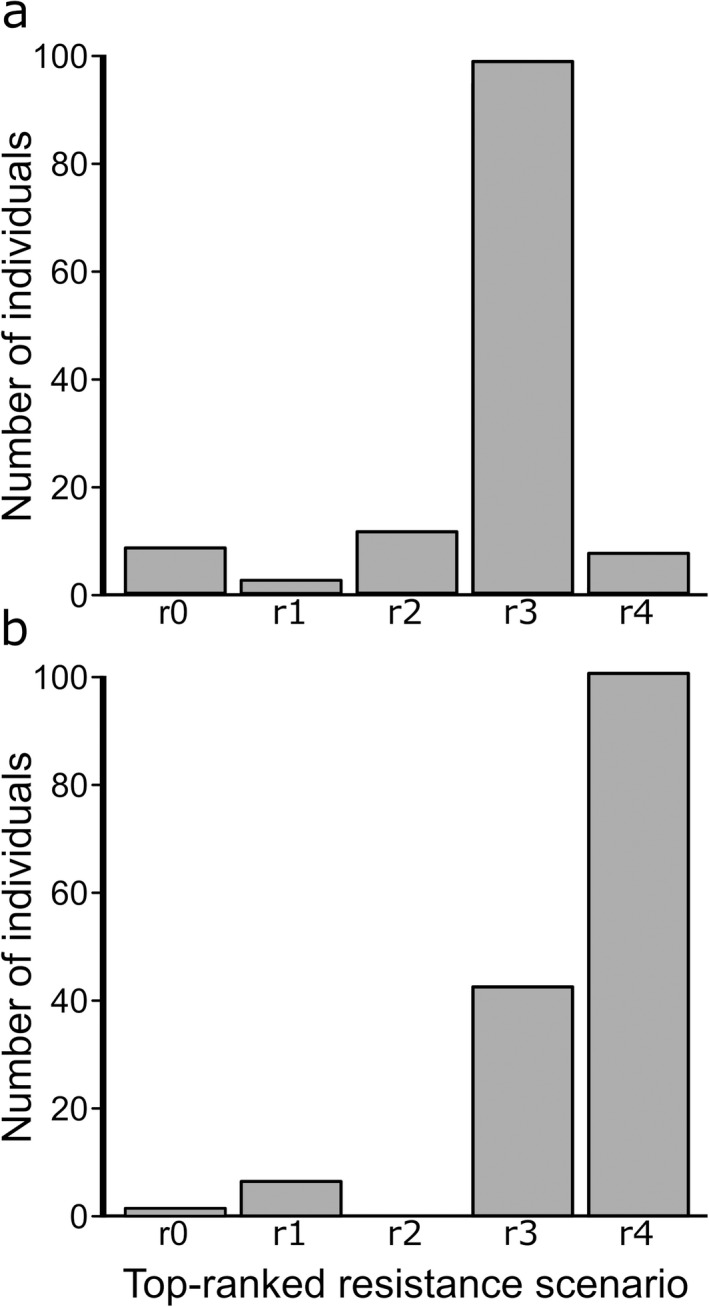
Distribution of top rankings among the five resistance scenarios considered for (a) fall and (b) spring migration. Scenarios with greater numbers of top rankings were considered to have greater relative support. An individual is defined here as the seasonal migration for a single caribou in a given year.

Considering resistance scenario rankings among individuals, support for different scenarios varied between individuals, but scenario r3 was the most common top‐ranked model for fall migration, occurring in about 75% of the top‐ranked models (Fig. 3a, Appendix S2: Table S6). In the spring, r3 featured in nearly 30% of the top‐ranked models, but was surpassed by r4, which was featured in 66% of the top‐ranked models (Fig. 3b, Appendix S2: Table S7). Scenario r2 was not ranked as a top model by any individual in the spring.

**Fig. 4:**
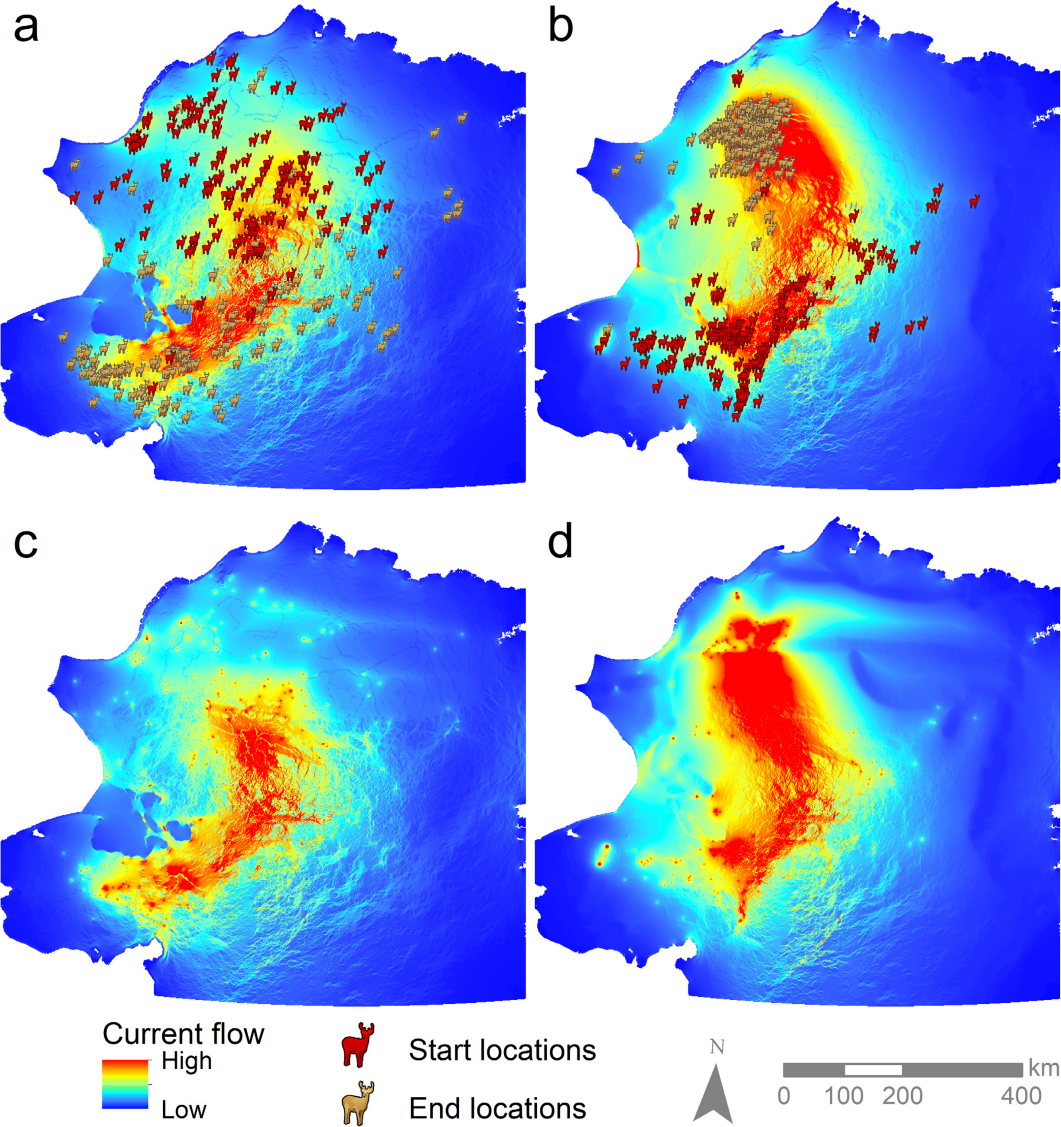
Population‐level migratory connectivity for the Western Arctic Herd in (a) fall with existing roads, (b) spring with existing roads, (c) fall with possible roads added, and (d) spring with possible roads added. Maps display summed current flow values under each individual’s top‐ranked model for the given season. Start and end locations are the same within seasons.

Population‐level connectivity maps for fall (Fig. 4a) and spring (Fig. 4b) migration revealed differences in the concentration of current flow between seasons. Both seasons showed similar areas of high connectivity in the south of the study area, though with greater intensity in spring than fall. Connectivity in the north differed across seasons, however, with spring featuring a strong concentration of high current flow and fall exhibiting more diffuse connectivity. *K*‐fold cross‐validation, reflecting how well population‐level current flow maps represent observed caribou locations, demonstrated high predictive ability of the models in both seasons ( ${\bar{\rho }}_{\text{fall}}=0.95$, ${\bar{\rho }}_{\text{spring}}=0.94$). Average connectivity values at observed caribou locations were higher than across the study area in both fall (mean ± SE at caribou locations: 6.6 × 10^−4^ ± 1.9 × 10^−5^, study area mean: 2.4 × 10^−4^) and spring (caribou locations: 9.1 × 10^−4^ ± 1.4 × 10^−5^, study area: 2.8 × 10^−4^).

**Fig. 5:**
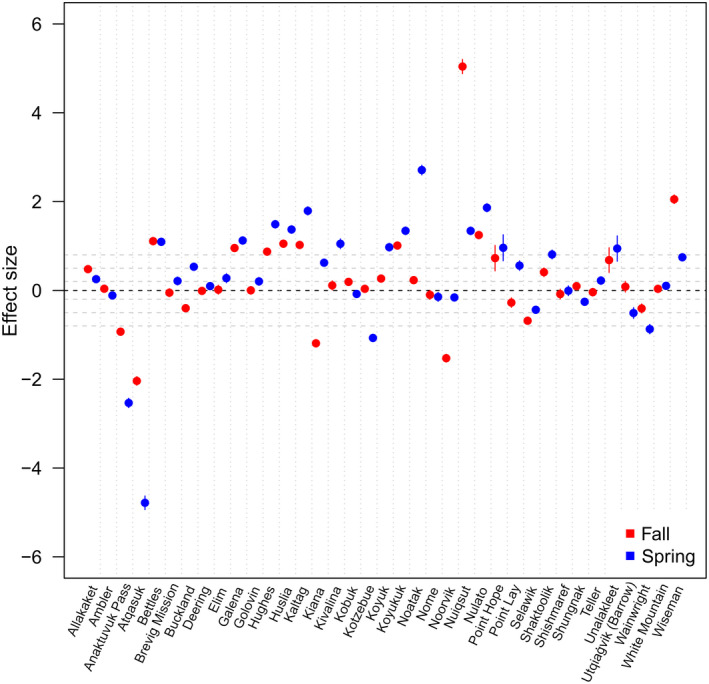
Estimated effect sizes and 95% confidence intervals for alteration in current flow of caribou near subsistence communities in northwestern Alaska in the presence of potential roads. Horizontal gray lines indicate bounds of the approximate effect levels proposed by Cohen (1992): abs(*d*) < 0.2, negligible; abs(*d*) < 0.5, small; abs(*d*) < 0.8, medium; abs(*d*) > 0.8, large. See Appendix S2: Fig. S6 for a map with all community locations and Fig. S7 for effect size results depicted spatially.

Adding a possible road system to the resistance landscape altered current flow patterns in both fall and spring, although overall patterns remained similar (Fig. 4c, d). Analysis of effect sizes indicated varying results for alteration of current flow near communities both over space and across seasons (Fig. 5). Communities in the southwest of the study area generally showed negligible change in current flow during fall migration with the addition of proposed roads, while those along the eastern periphery showed increased flow (Appendix S2: Fig. S7). Communities just to the east of the primary migration areas, as well as those to the north, showed decreases in current flow in fall. In spring, more communities showed increases in current flow compared to fall, though there were exceptions (e.g., Kotzebue, Shugnak) where current flow switched from expected increase to decrease across seasons.

## Discussion

Our analytical approach revealed broad‐scale landscape features that influenced the use of migration paths by caribou in northwestern Alaska. It further demonstrated potential impacts of a proposed road system on movement of a highly mobile species and the people who rely on it for hunting. Such understanding is vital to inform permitting decisions that balance new development, resource user needs, and the preservation of ecosystem function. Our methods are flexible and can easily be adapted to other species, locations, and development scenarios.

Caribou patterns of resource selection during migration generally aligned with relationships previously reported for the WAH. For example, the negative responses to rugged terrain, major rivers, and dense vegetation recorded in this study also were identified for WAH fall migration by Fullman et al. (2017). Dense vegetation, typically tall shrubs, may increase predation risk for caribou (Bergerud and Luttich 2003, Joly and Klein 2011, Pinard et al. 2012) and form a physical barrier to movement. The migratory period is a time of heightened wolf predation for the WAH (Ballard et al. 1997, Gurarie et al. 2020), favoring actions that reduce this risk. Avoidance of burned areas aligns with previous studies of caribou winter habitat use, where it has been related to reduced forage availability (Joly et al. 2003, 2007, Collins et al. 2011). The negative response of caribou to rugged terrain likely reflects increased energetic costs for migratory movements due to greater movement tortuosity (Wilson et al. 2013*a*) and the increased effort required to climb slopes (Fancy and White 1987), though studies have shown that caribou select more varied topography at fine scales (Joly 2011, 2011, Wilson et al. 2014) due to increased forage availability (Nellemann and Thomsen 1994, Nellemann and Fry 1995).

Selection of areas farther from existing roads was clearly indicated across both seasons, despite the paucity of roads in the study area. Multiple studies have found that caribou and reindeer may show avoidance or altered movement behavior in proximity to roads (Leblond et al. 2013, Panzacchi et al. 2013, Wilson et al. 2016, Baltensperger and Joly 2019) and that road effects may extend beyond their physical footprint due to noise, dust, human activity, and other factors (Myers‐Smith et al. 2006, Shannon et al. 2014, Paton et al. 2017). While our study finds a negative response to roads during migration, this does not imply that roads pose an absolute barrier. Future studies should quantify both the degree of permeability of roads for caribou (Beyer et al. 2016) and drivers of permeability (e.g., landscape context, road type, traffic), as these factors likely have a strong influence on the overall impact of roads on caribou (Muhly et al. 2015). Other potential concerns also need to be investigated, such as the possibility of roads increasing vulnerability to predators (Whittington et al. 2011, Bojarska et al. 2017, Newton et al. 2017). Additionally, there may be road density threshold effects where the cumulative impacts of additional roads could result in the failure of migration altogether.

Results for caribou subsistence hunters under a realistic road system proposal were mixed. While the overall pattern of caribou connectivity remained similar (Fig. 4), our models predicted altered current flow with the addition of proposed roads that led to reductions in caribou flow for several communities near the center of current migration areas (Appendix S2: Fig. S7). These predictions should receive careful consideration as new projects are being planned to ensure that resource development opportunities do not end up undermining subsistence opportunities, as has been suggested in other countries (Parlee et al. 2018). Some communities may be able to substitute reductions in caribou availability with other resources (e.g., marine mammals), while for others that are highly dependent on caribou the impacts may be felt more strongly (Anaktuvuk Pass; Bacon et al. 2011, Martin 2015). It is possible that changes in caribou movement patterns in response to new development may increase subsistence opportunities for some communities, as is suggested for communities in the southern and eastern portions of the study area. However, the effects of new roads are likely to be complicated. They may increase access for subsistence hunting, potentially compensating for altered caribou flow near communities. At the same time, they may facilitate access by non‐local hunters, which could reduce subsistence harvest success (Guettabi et al. 2016). In northwest Alaska, there are already reports of user conflict between hunters from within and outside of the region (Georgette and Loon 1988, Fix and Harrington 2012, Halas 2015). Hunting around potential roads may compound road effects on species movement, leading to increased avoidance of roads by caribou (Paton et al. 2017, Plante et al. 2018). Furthermore, other future development such as the Ambler Road, which is currently undergoing permitting for access to the Ambler Mining District and is proposed to run through the southeastern part of the study area (BLM 2019), could result in cumulative effects when combined with the possible roads evaluated in our analysis, altering the consequences for communities in the region. The potential for cumulative effects, altered access and increased user conflict should be considered in future analyses of any road proposals.

Our findings are not the final word on impacts from a possible road system in the area. Planning for the ASTAR project is ongoing and community consultations have yet to be held in many places. The routes and lengths of proposed roads have already been modified multiple times and we expect there will be further modifications prior to any roads being built. Furthermore, analysis of potential road impacts on caribou is just one of many analyses that would need to be conducted prior to a road project being developed. Mitigation measures (e.g., reduced speed limits, closures during migration, and over‐ or underpasses) may further alter resistances experienced by caribou relative to our analysis, although impacts to migratory movements were still noted in the presence of mitigation measures along the road to the Red Dog Mine within our study area (Wilson et al. 2016). Finally, full evaluation of potential impacts of road system proposals should consider effects on wildlife species during their complete annual cycle. For example, in the road system proposal we evaluated, the WAH calving area is bisected by three roads. Sensitivity of calving caribou and those with young calves has been noted around oil and gas development in other parts of northern Alaska (Griffith et al. 2002, Cameron et al. 2005, Joly et al. 2006). Methods for analyzing potential development impacts on habitat selection of caribou in other seasons already exist (Wilson et al. 2013*b*, 2014) and should be complemented by our migration‐focused approach, or other similar methods, to provide a more robust representation of the cumulative effects of potential road systems.

### Utility of our approach

We applied circuit theory to examine broad‐scale selection in the context of entire migration pathways and to examine the relationship between landscape resistance and relative habitat suitability. For fall migration, a negative linear relationship between habitat suitability and landscape resistance was strongly supported for most caribou (Fig. 3a, Appendix S2: Table S6), aligning with one of the most commonly used approaches to deriving resistance surfaces from resource selection studies (Zeller et al. 2012). The different pattern observed for spring migration, with about two‐thirds of caribou migrations indicating the negative logarithmic as their top‐ranked resistance model (Fig. 3b, Appendix S2: Table S7), reinforces the importance of incorporating season‐specific resistance rasters (Cushman and Lewis 2010, Mui et al. 2017). The strength of resistance relationships can change across seasons, even if the patterns of RSF coefficients remain similar.

While this study focused on caribou, our approach can readily be applied to other migratory species. In addition, our approach is applicable beyond migration and can be used to understand selection during other types of directed movement in which start and end locations are known and spatial data are available along the movement route. As such, circuit theory provides the opportunity to expand our understanding of multiple movement behaviors and to test hypotheses about how landscape resistance relates to habitat selection.

Myriad studies exist analyzing effects of existing roads and other development on wildlife connectivity (Lendrum et al. 2012, Ito et al. 2013, Prokopenko et al. 2017, Plante et al. 2018, Quaglietta et al. 2019), but fewer consider the effects of future or proposed roads. Most studies that do consider proposed roads utilize simulation models (Finke et al. 2008, Holdo et al. 2011, Davey et al. 2017). We provide an empirically based analytical approach to assessing proposed road impacts using animal movement data. Genetic analyses provide an alternative approach to assess landscape resistance and development impacts (Epps et al. 2007). In situations where this type of information is not available, our approach provides a means to identify movement resistance and development impacts.

### Conservation and policy implications

Disruption of migration routes has at times led to sudden and severe population declines for migratory species (Bolger et al. 2008). The consequences of disrupted migrations may ripple out to negatively affect other ecosystem components, such as predators that rely on migratory species (Walton et al. 2017) or alternative prey species that face increased pressure from predators when migrants are removed (Lee et al. 2016). In places like the Arctic, disrupted migration could also affect subsistence harvests. It is thus crucial to conduct development projects in a way that minimizes hindrances to migration. When making decisions, it is necessary to decide when adverse impacts are considered acceptable and when they should lead to restrictions on, or mitigation of, activities (Gende et al. 2018). Examining potential effects of roads and other developments before they are constructed is an important step in this direction, facilitating informed discussions with stakeholders and communities about the advantages and disadvantages of alternative proposals. Our approach, using circuit theory to evaluate potential impacts of proposed development while simultaneously selecting between alternative landscape resistance scenarios, presents one technique to better inform such discussions and planning.

## Supporting information

Appendix S1

Appendix S2

Data S1

## Footnotes

^8^
http://nhd.usgs.gov
