# Supplementary material for: Mapping potential effects of proposed roads on migratory connectivity for a highly mobile herbivore using circuit theory
Source: Ecol Appl. 2020 Aug 18;31(1):e2207. doi: 10.1002/eap.2207 (PMC7816249; doi:10.1002/eap.2207)
Supplement: Supplementary file 1 — Appendix S1 [file EAP-31-e2207-s001.pdf]

Supporting Information. Fullman, T.J., R.R. Wilson, K. Joly, D.D. Gustine, P. Leonard, and W.M. Loya. 2020. Mapping potential effects of proposed roads on migratory connectivity for a highly mobile herbivore using circuit theory. Ecological Applications.

Appendix S1: Availability sample size sensitivity analysis

We conducted a sensitivity analysis on the effects of the size of the availability sample on our resource selection function (RSF) coefficient estimates following the guidance of Northrup et al. (2013). For each used (i.e., observed) caribou location, a candidate set of 200 potentially available locations was randomly generated across the study area (see Appendix S2: Figure S1 for study area extent) and environmental covariate values were sampled at the observed and available candidate locations. We tested seven availability ratios ranging from 1 to 50 available locations per used location (availability ratios = 1, 3, 5, 10, 20, 35 and 50). We fit RSF models using the full model (Model 15 in Appendix S2: Tables S4 – S5) with available points randomly selected from the candidate set. This was repeated 100 times for each availability ratio. Using the results, we calculated the mean coefficient estimate and 95% simulation envelope. We ran separate sensitivity analyses for fall and spring migration, each following the procedure above.

Simulation results indicated relatively small changes in regression coefficient estimates across varying availability ratios (Figures S1 – S2). The sign of all coefficients remained the same for each availability ratio. Simulation envelopes grew narrower as the availability ratio increased, and coefficient estimates tended to stabilise at around 20 available locations per used location. These effects were consistent across both fall and spring migration. In light of these results, we randomly subset the candidate availability data to yield a final availability ratio of 20 available locations for each used location in subsequent analyses.

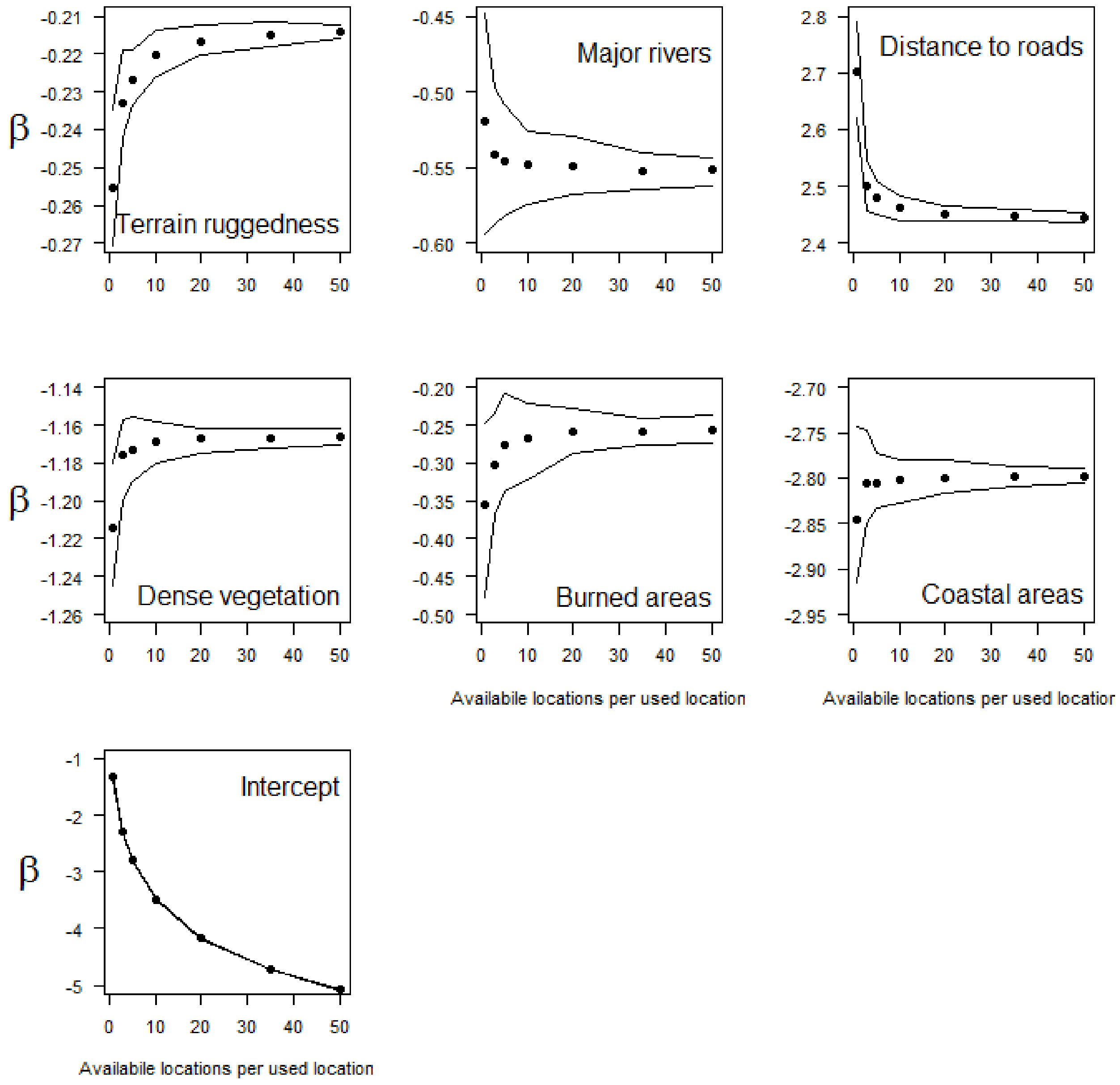

Figure S1. Fall migration coefficient estimates (black points) and 95% simulation envelopes (solid lines) for Western Arctic Herd selection of environmental covariates, calculated from 100 resource selection function models fit for varying ratios of available to used locations. Distance to roads was included as an exponential distance decay function of the form $e^{-\alpha/d}$, where d is the distance from each used or available location to the nearest road in kilometers and α varied between seasons (α = 60 for fall migration, see main text for details). Note that y-axes vary across panels.

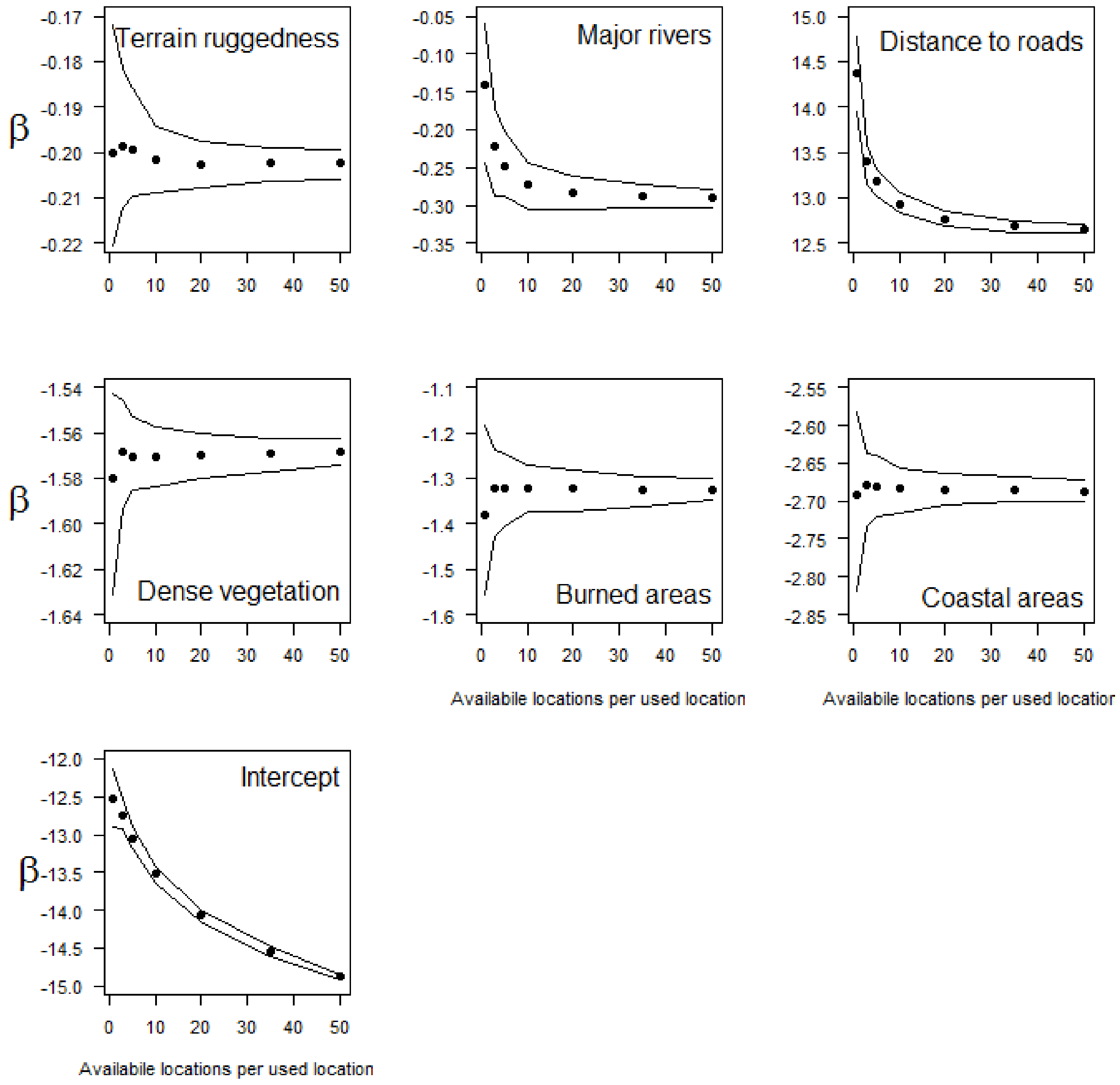

Figure S2. Spring migration coefficient estimates (black points) and 95% simulation envelopes (solid lines) for Western Arctic Herd selection of environmental covariates, calculated from 100 resource selection function models fit for varying ratios of available to used locations. Distance to roads was included as an exponential distance decay function of the form $e^{-\alpha/d}$, where d is the distance from each used or available location to the nearest road in kilometers and α varied between seasons (α = 14 for spring migration, see main text for details). Note that y-axes vary across panels.

References

Northrup, J. M., M. B. Hooten, C. R. Anderson Jr., and G. Wittemyer. 2013. Practical guidance on characterizing availability in resource selection functions under a use-availability design. Ecology 94:1456–1463.
