# Supplementary material for: Mapping potential effects of proposed roads on migratory connectivity for a highly mobile herbivore using circuit theory
Source: Ecol Appl. 2020 Aug 18;31(1):e2207. doi: 10.1002/eap.2207 (PMC7816249; doi:10.1002/eap.2207)
Supplement: Supplementary file 2 — Appendix S2 [file EAP-31-e2207-s002.pdf]

Supporting Information. Fullman, T.J., R.R. Wilson, K. Joly, D.D. Gustine, P. Leonard, and W.M. Loya. 2020. Mapping potential effects of proposed roads on migratory connectivity for a highly mobile herbivore using circuit theory. Ecological Applications.

Appendix S2: Additional tables and figures

Table S1. Land cover reclassification crosswalk table depicting relationships between the coarse classes of Boggs et al. (2014) and the classes used in our analysis.

Our classification	Boggs et al. (2014) classification
Open areas	Tundra<br>Low shrub<br>Barren ground<br>Freshwater<br>Perennial ice and snow
Dense vegetation	Forest<br>Riparian<br>Tall shrub<br>Dense shrub
Burned areas	Burned
Coastal waters	Not in Boggs et al. (2014). This consisted of all offshore areas within 3 km of the coast, plus all of Kotzebue Sound.

See Appendix S2: Figure S1 for a depiction of the land cover classes used in our analysis.

Table S2. Road response model selection results for Western Arctic Herd fall migration.

Road response [1]	K [2]	Δ AICc [3]	Akaike Weight	Log-Likelihood
α = 60	4	0.00	0.87	-64726
α = 50	4	3.76	0.13	-64728
α = 45	4	19.83	0.00	-64736
α = 70	4	22.76	0.00	-64737
α = 40	4	48.80	0.00	-64750
α = 80	4	63.25	0.00	-64758
α = 35	4	94.47	0.00	-64773
α = 90	4	115.90	0.00	-64784
α = 30	4	162.23	0.00	-64807
α = 100	4	177.02	0.00	-64815
α = 25	4	260.08	0.00	-64856
α = 20	4	400.50	0.00	-64926
α = 19	4	435.31	0.00	-64944
α = 18	4	472.84	0.00	-64962
α = 17	4	513.35	0.00	-64983
α = 150	4	541.48	0.00	-64997
α = 16	4	557.12	0.00	-65005
α = 15	4	604.52	0.00	-65028
α = 14	4	655.92	0.00	-65054
α = 13	4	711.79	0.00	-65082
α = 12	4	772.69	0.00	-65112
α = 11	4	839.28	0.00	-65146
α = 10	4	912.37	0.00	-65182
α = 200	4	924.67	0.00	-65188
α = 9	4	992.99	0.00	-65222
α = 8	4	1082.42	0.00	-65267
α = 7	4	1182.37	0.00	-65317
α = 250	4	1286.26	0.00	-65369
α = 6	4	1295.20	0.00	-65374
linear	4	1311.66	0.00	-65382
α = 5	4	1424.28	0.00	-65438
α = 4	4	1574.80	0.00	-65513
α = 300	4	1615.50	0.00	-65534
α = 3	4	1755.69	0.00	-65604
α = 350	4	1910.93	0.00	-65681
α = 2	4	1985.59	0.00	-65719
α = 1	4	2328.22	0.00	-65890

[1] linear = road response included as a simple linear function of distance to road in kilometers. α = road effects included as an exponential distance decay function, $e^{-\alpha/d}$, where d = distance from each location to the nearest road in kilometers. Different values of α were compared for each season. See main text for details.

[2] K = number of parameters in the model.

[3] AICc = Akaike's Information Criterion adjusted for small sample size.

Table S3. Road response model selection results for Western Arctic Herd spring migration.

Road response [1]	K [2]	Δ AICc [3]	Akaike Weight	Log-Likelihood
α = 14	4	0.00	0.25	-48871
α = 13	4	0.05	0.24	-48871
α = 15	4	0.76	0.17	-48871
α = 12	4	1.02	0.15	-48872
α = 16	4	2.25	0.08	-48872
α = 11	4	3.04	0.05	-48873
α = 17	4	4.41	0.03	-48873
α = 10	4	6.30	0.01	-48874
α = 18	4	7.18	0.01	-48875
α = 19	4	10.54	0.00	-48876
α = 9	4	11.06	0.00	-48877
α = 20	4	14.46	0.00	-48878
α = 8	4	17.67	0.00	-48880
α = 7	4	26.67	0.00	-48884
α = 6	4	38.84	0.00	-48890
α = 25	4	41.65	0.00	-48892
α = 5	4	55.40	0.00	-48899
α = 4	4	71.25	0.00	-48907
α = 3	4	76.21	0.00	-48909
α = 2	4	78.06	0.00	-48910
α = 30	4	80.45	0.00	-48911
α = 1	4	80.49	0.00	-48911
α = 35	4	129.72	0.00	-48936
α = 40	4	188.36	0.00	-48965
α = 45	4	255.24	0.00	-48999
α = 50	4	329.19	0.00	-49036
α = 60	4	494.15	0.00	-49118
α = 70	4	675.89	0.00	-49209
α = 80	4	868.78	0.00	-49305
α = 90	4	1068.60	0.00	-49405
α = 100	4	1272.29	0.00	-49507
α = 150	4	2286.44	0.00	-50014
α = 200	4	3220.99	0.00	-50482
α = 250	4	4046.22	0.00	-50894
linear	4	4296.85	0.00	-51019
α = 300	4	4763.40	0.00	-51253
α = 350	4	5382.35	0.00	-51562

[1] linear = road response included as a simple linear function of distance to road in kilometers. α = road effects included as an exponential distance decay function, $e^{-\alpha/d}$, where d = distance from each location to the nearest road in kilometers. Different values of α were compared for each season. See main text for details.

[2] K = number of parameters in the model.

[3] AICc = Akaike's Information Criterion adjusted for small sample size.

Table S4. Model selection results for Western Arctic Herd resource selection during fall migration.

Model	Parameters [1]	K [2]	Δ AICc [3]	Akaike Weight	Log-Likelihood
15	rug, riv, rd, lc	9	0	1	-62407
13	rug, rd, lc	8	199	0	-62508
14	riv, rd, lc	8	516	0	-62666
10	rd, lc	7	670	0	-62744
11	rug, riv, rd	6	4120	0	-64470
6	rug, rd	5	4325	0	-64574
12	rug, riv, lc	8	4376	0	-64596
8	riv, rd	5	4458	0	-64640
3	rd	4	4628	0	-64726
7	rug, lc	7	4640	0	-64729
9	riv, lc	7	5154	0	-64986
4	lc	6	5354	0	-65087
5	rug, riv	5	8207	0	-66515
1	rug	4	8465	0	-66645
2	riv	4	8713	0	-66769
0	int	3	8922	0	-66874

[1] rug = terrain ruggedness, riv = major rivers, rd = existing roads, lc = land cover, int = intercept-only.

[2] K = number of parameters in the model.

[3] AICc = Akaike's Information Criterion adjusted for small sample size.

Table S5. Model selection results for Western Arctic Herd resource selection during spring migration.

Model	Parameters [1]	K [2]	Δ AICc [3]	Akaike Weight	Log-Likelihood
15	rug, riv, rd, lc	9	0	1	-46427
13	rug, rd, lc	8	43	0	-46450
14	riv, rd, lc	8	332	0	-46594
10	rd, lc	7	358	0	-46608
11	rug, riv, rd	6	4627	0	-48744
6	rug, rd	5	4679	0	-48771
8	riv, rd	5	4840	0	-48851
3	rd	4	4877	0	-48871
12	rug, riv, lc	8	8868	0	-50862
7	rug, lc	7	8959	0	-50909
9	riv, lc	7	9592	0	-51226
4	lc	6	9647	0	-51254
5	rug, riv	5	13167	0	-53015
1	rug	4	13257	0	-53061
2	riv	4	13619	0	-53242
0	int	3	13680	0	-53273

[1] rug = terrain ruggedness, riv = major rivers, rd = existing roads, lc = land cover, int = intercept-only.
[2] K = number of parameters in the model.
[3] AICc = Akaike's Information Criterion adjusted for small sample size.

Table S6. Consistency of support among top-ranked models for the various resistance scenarios compared for Western Arctic Herd fall migration.

Scenario [1]	n [2]	%
r0	9	6.87
r1	3	2.29
r2	12	9.16
r3	99	75.57
r4	8	6.11

[1] See Table 2 in the main text for descriptions and formulas of resistance scenarios.
[2] Number of individuals for which this was their top-ranked model during fall migration. An individual is defined here as the seasonal migration for a single caribou in a given year.

Table S7. Consistency of support among top-ranked models for the various resistance scenarios compared for Western Arctic Herd spring migration.

Scenario [1]	n [2]	%
r0	2	1.31
r1	7	4.58
r2	0	0.00
r3	43	28.10
r4	101	66.01

[1] See Table 2 in the main text for descriptions and formulas of resistance scenarios.
[2] Number of individuals for which this was their top-ranked model during spring migration. An individual is defined here as the seasonal migration for a single caribou in a given year.

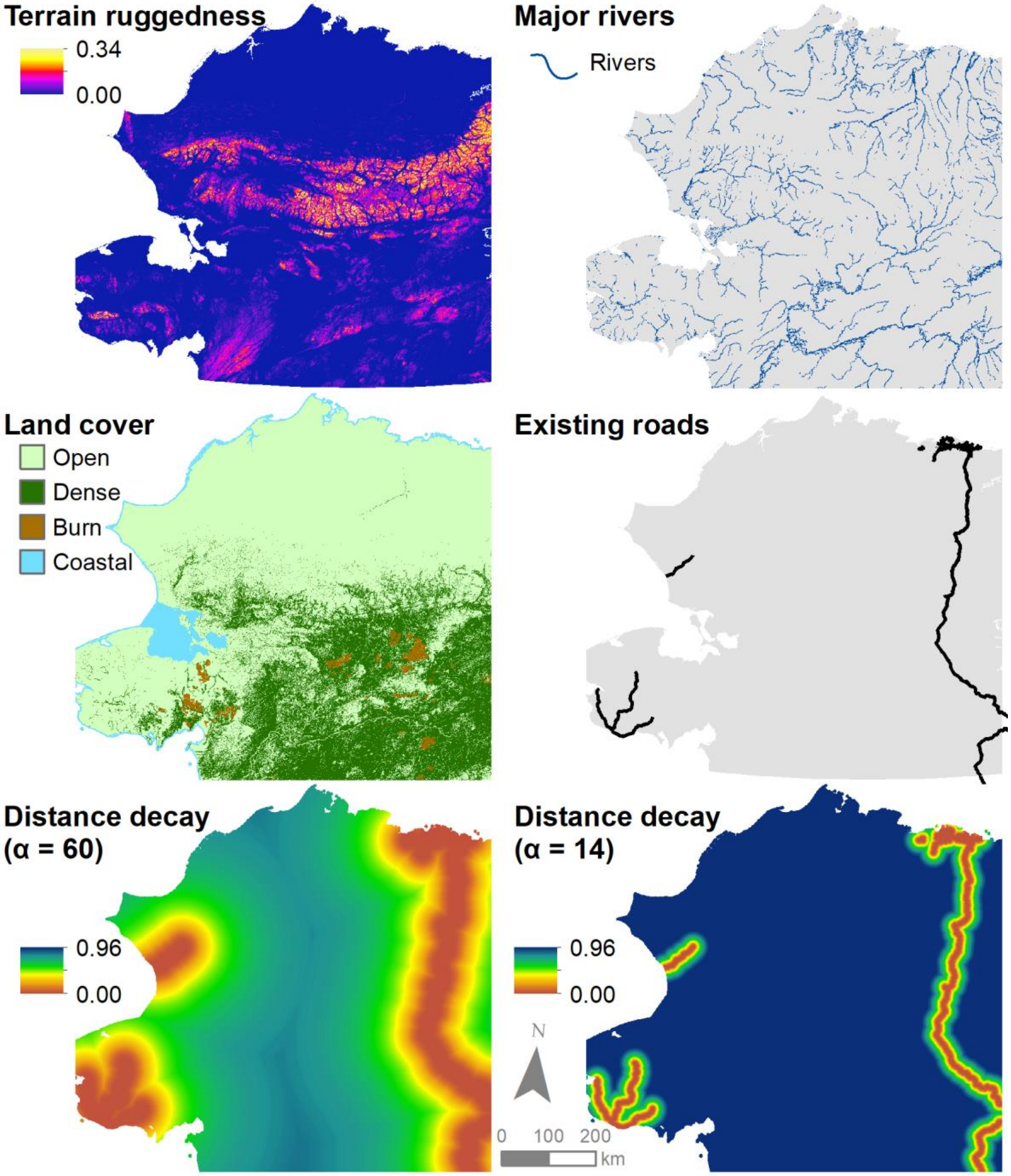

Figure S1. Environmental variables analyzed for Western Arctic Herd selection and landscape resistance during fall and spring migration. Land cover classes: Open = open areas, Dense = dense vegetation, Burn = burned areas, Coastal = coastal waters. See Appendix S2: Table S1 for land cover reclassification crosswalk. Distance to existing roads was included in the models using season-specific exponential decay functions of the form $e^{-\alpha/d}$, where d is the distance to the nearest road in kilometers and α varied between seasons. For fall migration α = 60 while for spring migration α = 14, based on model selection analyses (see main text for details).

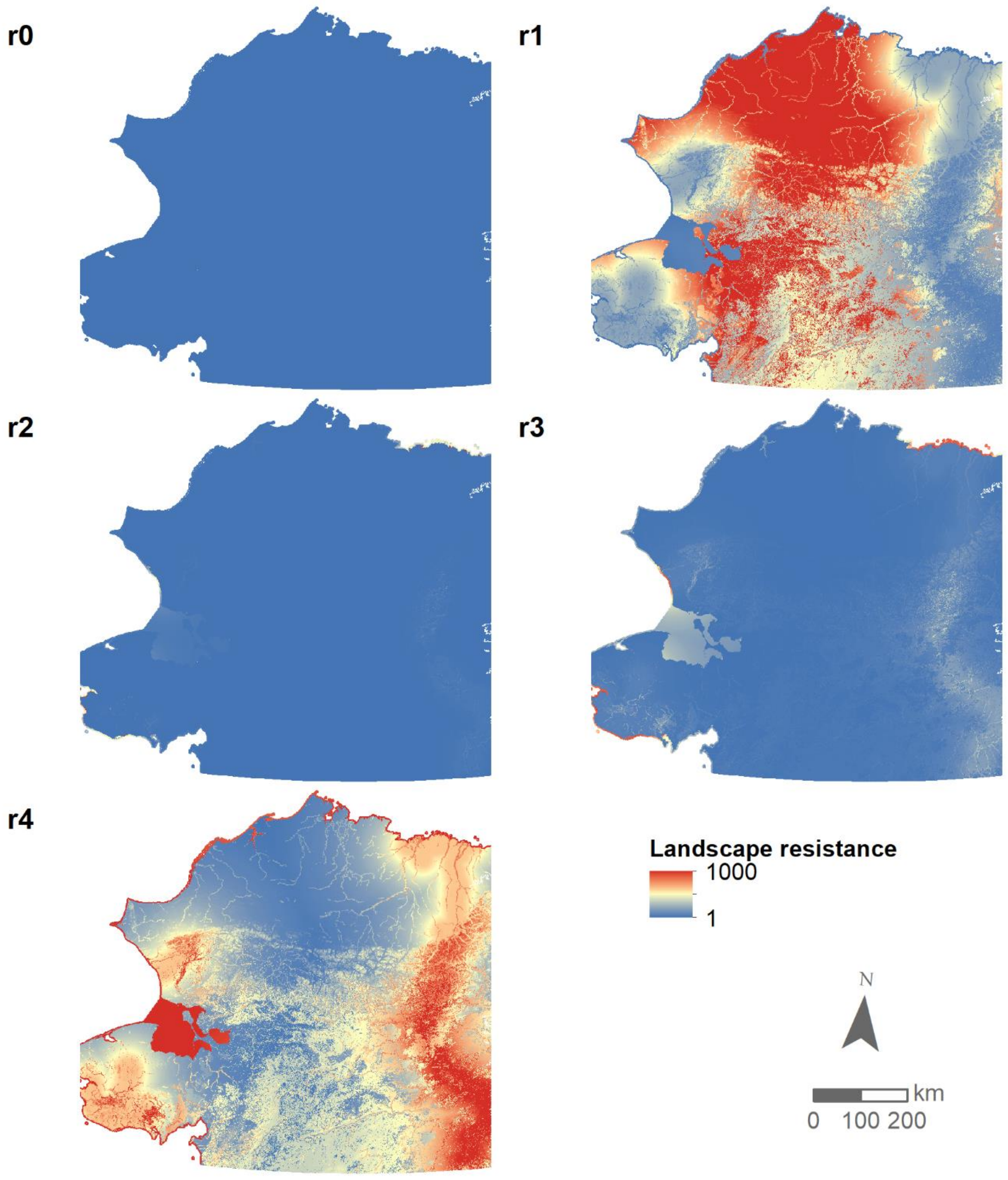

Figure S2. Landscape resistance scenarios evaluated for Western Arctic Herd fall migration under existing roads. See Table 2 in the main text for resistance scenario descriptions and formulas.

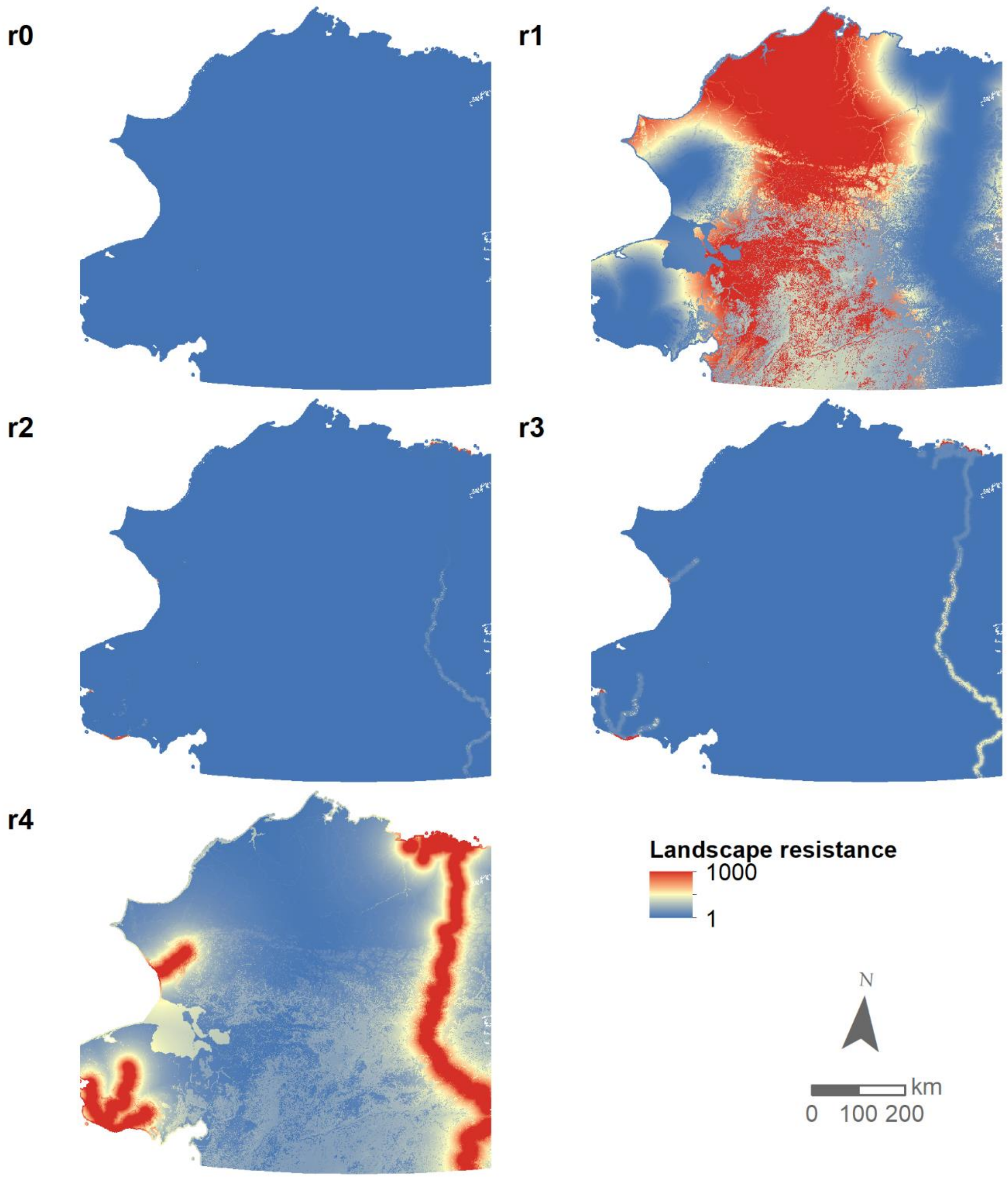

Figure S3. Landscape resistance scenarios evaluated for Western Arctic Herd spring migration under existing roads. See Table 2 in the main text for resistance scenario descriptions and formulas.

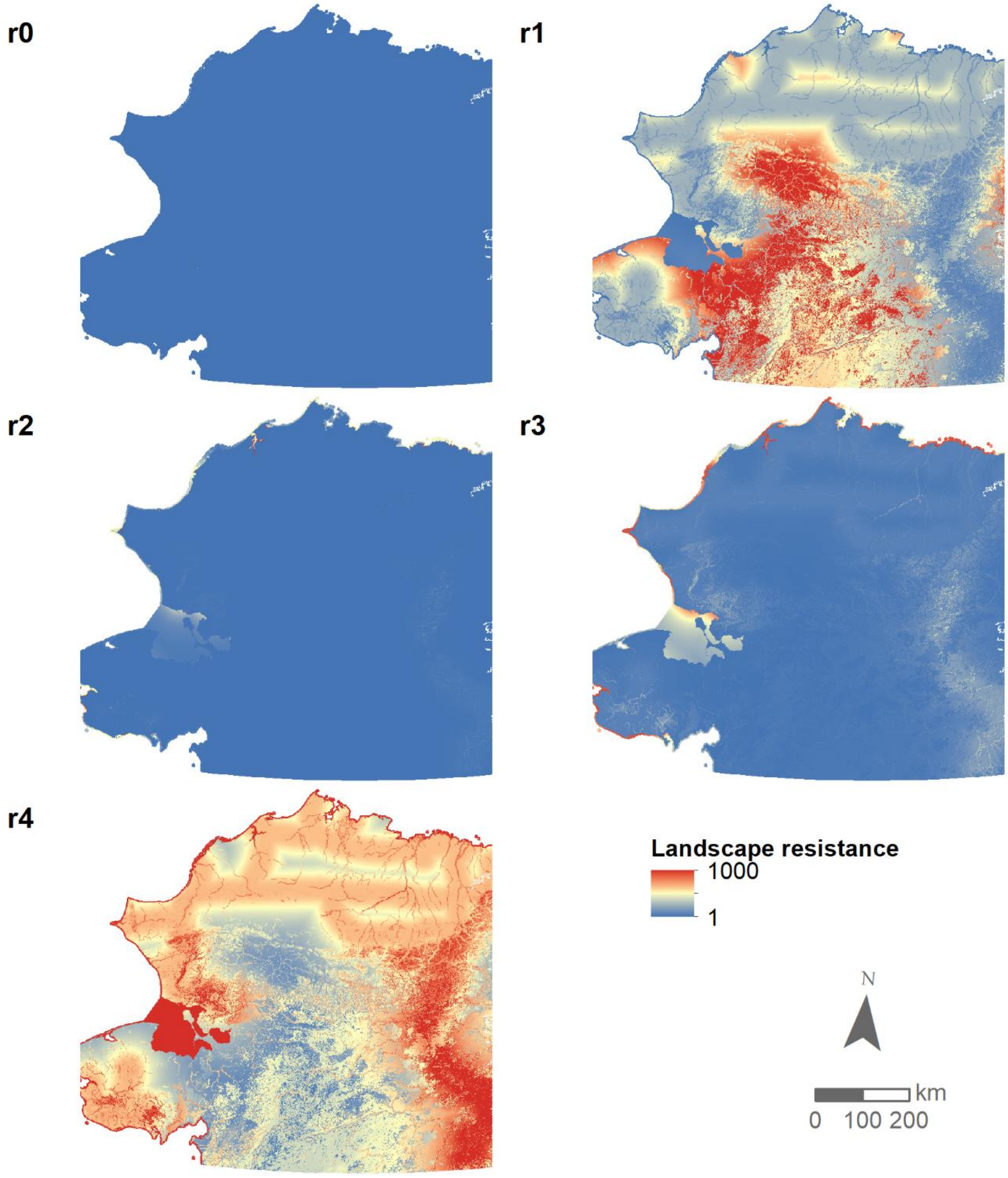

Figure S4. Landscape resistance scenarios evaluated for Western Arctic Herd fall migration with possible Arctic Strategic Transportation and Resources (ASTAR) roads added. See Table 2 in the main text for resistance scenario descriptions and formulas.

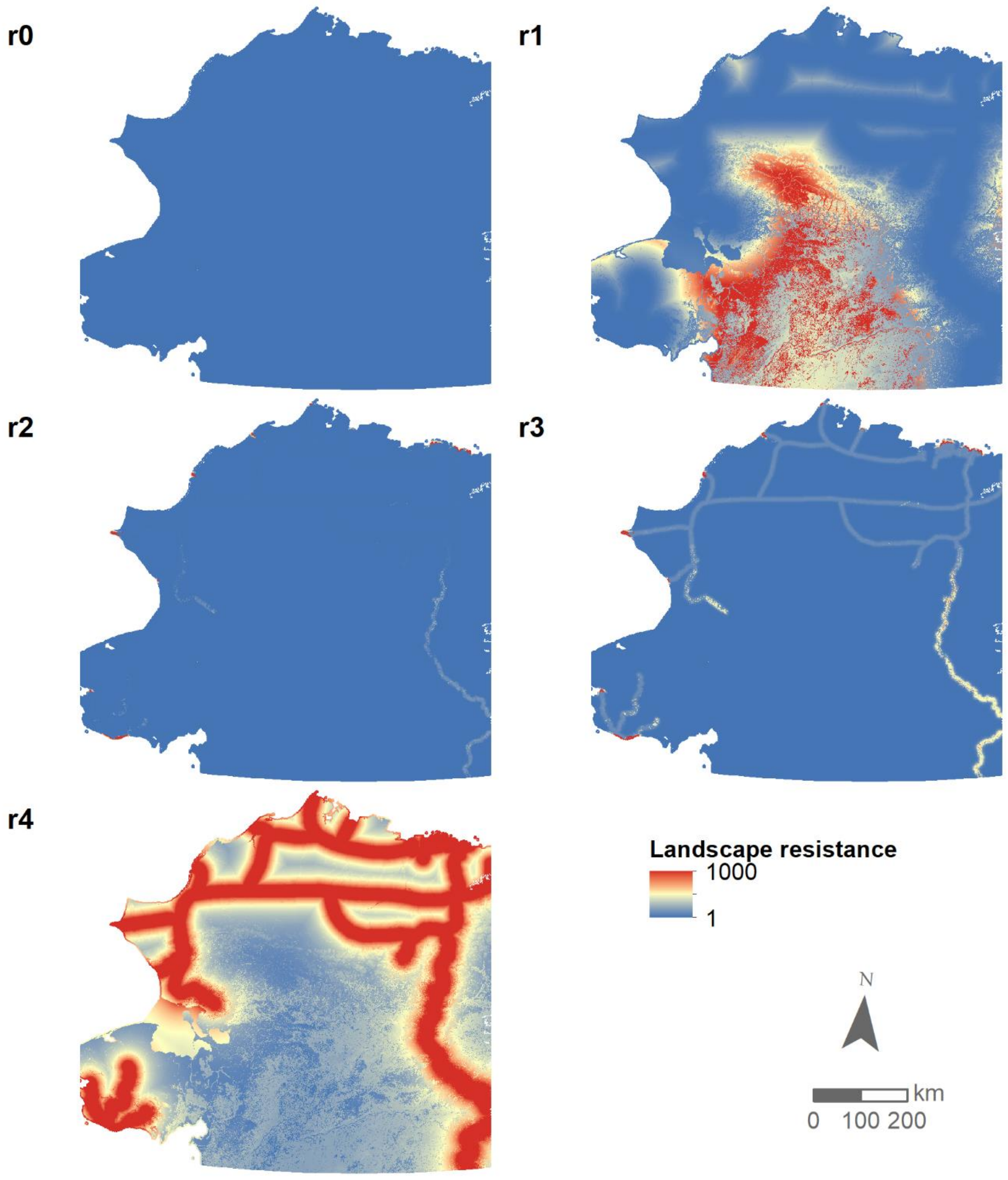

Figure S5. Landscape resistance scenarios evaluated for Western Arctic Herd spring migration with possible Arctic Strategic Transportation and Resources (ASTAR) roads added. See Table 2 in the main text for resistance scenario descriptions and formulas.

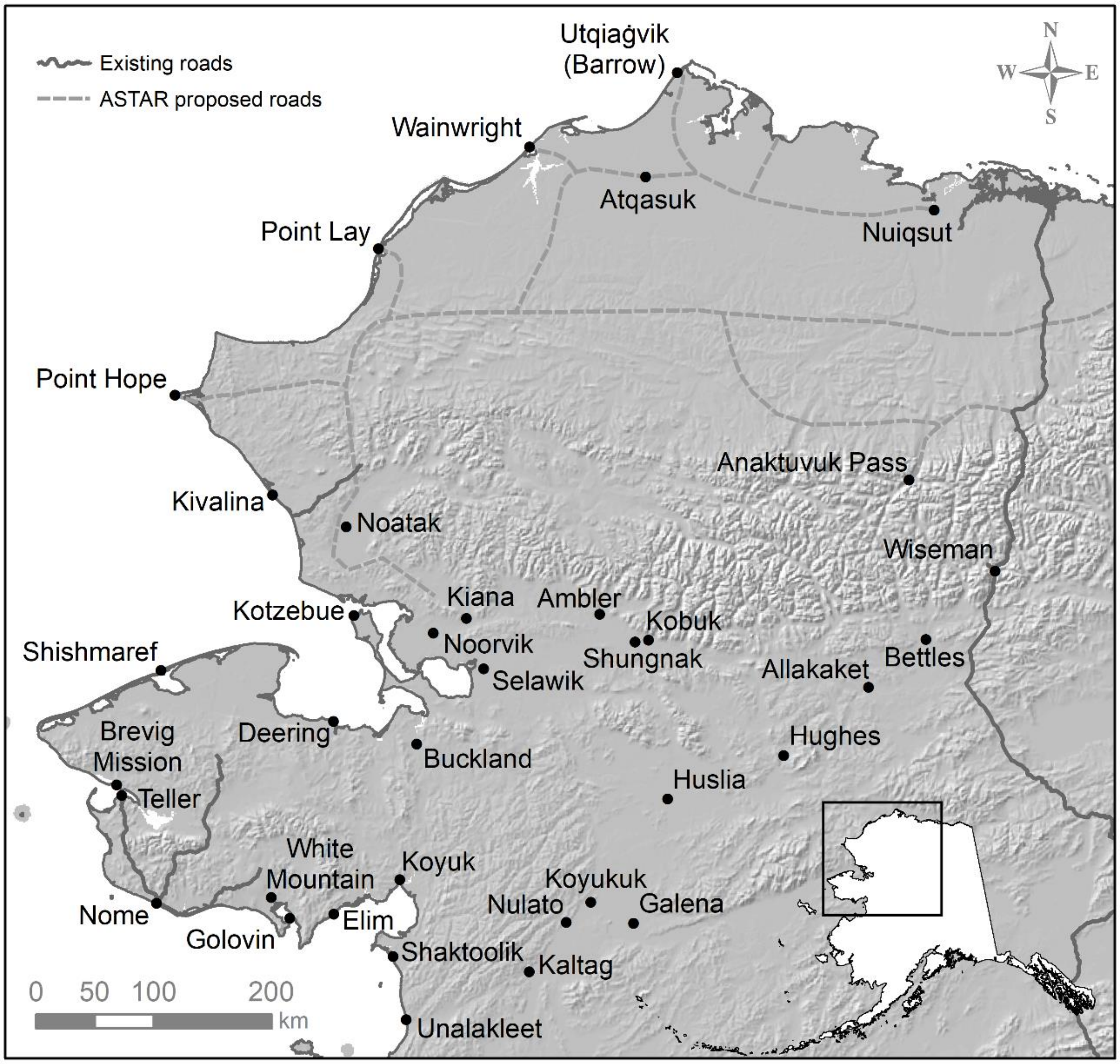

Figure S6. Communities analyzed with respect to changes in current flow value in the presence of possible Arctic Strategic Transportation and Resources (ASTAR) roads.

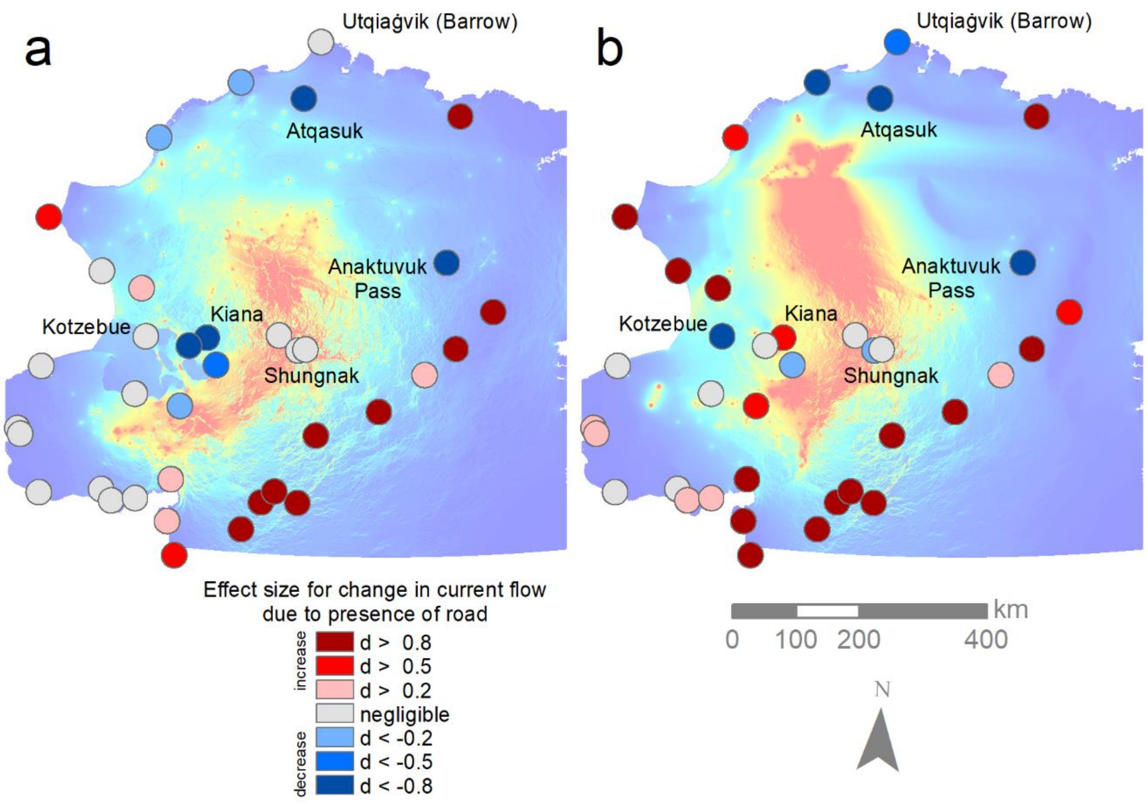

Figure S7. Predicted changes in current flow of caribou near subsistence communities in northwestern Alaska in the presence of potential roads during (a) fall migration and (b) spring migration. Circles indicate the area within 20 km of each community in which changes in current flow were evaluated. Colors represent estimated effect sizes for the change in current flow value around each community using raw score standardization. Thresholds of potential effects follow Cohen (1992). For a map with all communities labelled see Appendix S2: Figure S6.

References

Boggs, K., T. V Boucher, T. T. Kuo, D. Fehringer, and S. Guyer. 2014. Vegetation Map and Classification: Northern, Western and Interior Alaska. Alaska Center for Conservation Science (formerly Alaska Natural Heritage Program), University of Alaska Anchorage, Anchorage, AK, USA. Available from: http://accs.uaa.alaska.edu/vegetation-ecology/vegetation-map-northern-western-and-interior-alaska/. Accessed October 2014.

Cohen, J. 1992. A power primer. Psychological Bulletin 112:155–159.
